# The first transcriptome dataset of roselle (*Hibiscus sabdariffa* L.*)* calyces during maturation

**DOI:** 10.1016/j.dib.2022.108613

**Published:** 2022-09-17

**Authors:** Nur Atheeqah Hamzah, Christina Seok Yien Yong, Meenakshii Nallappan

**Affiliations:** Department of Biology, Universiti Putra Malaysia, Jalan Universiti 1, 43400 UPM Serdang, Selangor Darul Ehsan, Malaysia

**Keywords:** Anthocyanins, Phytochemicals, Differential gene expression, *Hibiscus sabdariffa* var UMKL, Secondary metabolites, RNA sequencing

## Abstract

Roselle (*Hibiscus sabdariffa* L.) is recognized for its phytochemical compounds such as anthocyanins, which possess pharmacological potentials in the treatments of hypertension, diabetes, cancer, hyperlipidaemia and hyperglycaemia. The calyx is the most commercially valuable part of the roselle and usually harvested at maturation. However, genetic study to understand the transcriptome changes in the calyx during maturation has yet to be explored. In this study, we sequenced the transcriptomes of roselle calyces at maturation stages III and IV using Illumina NextSeq 500 platform. These are the two most critical maturation stages in roselle, as these stages are often associated with the quality of the calyx. Over 200 million good quality paired-end reads were generated and *de novo* assembled into a reference transcriptome consisting of 221,334 transcripts with N50 score of 491bp. Among these transcripts, 92,974 transcripts (42%) were successfully annotated. The total number of significantly differentially expressed genes (DEGs) and the top five most significantly regulated genes in each of the maturation stage were presented. Twenty-one genes implicated in the biosynthesis of anthocyanins and their relative expressions in the calyx tissues at the two maturation stages were reported. Two secondary metabolites biosynthesis pathways that attained a relatively higher number of DEG mappings compared to other pathways were also reported. The findings from this work provide novel insights to better understand the transcriptional changes in roselle during calyx maturation, and the data made available here is intended for continued genetic study on roselle. The work is registered under NCBI Bioproject PRJNA664826. The raw sequencing reads are available in Short Read Archive with the accession numbers SRX9171161, SRX9171162, SRX9171163, SRX9171164, SRX9171165 and SRX9171166.

## Specifications Table

**Table:** 

Subject	Biological Sciences
Specific subject area	Omics: Transcriptomics
Type of data	Sequencing raw reads, assembly statistics, table, graph, Fig..
How the data were acquired	The data was obtained via a 76-bp paired end sequencing using the Illumina NextSeq 500 sequencer.
Data format	Raw reads (FASTQ format)
Description of data collection	Samples were collected at two maturation stages of the calyces, the stage III and stage IV maturation stages. Total RNA was extracted from calyx tissue samples using RNeasy Plant Mini Kit (Qiagen, Hilden, Germany) and purified prior to rRNA-depletion using Terminator™ 5’-Phosphate-Dependant Exonuclease (Epicentre, Madison, USA). The constructions of cDNA sequencing libraries were carried out using ScriptSeq™ v2 RNA-Seq Library Preparation Kit (Epicentre, Madison, USA) and sequenced using Illumina NextSeq 500 Sequencing platform (Illumina, USA), which generated 76 bp paired-end sequences. Three sequencing libraries were constructed for each maturation stage.
Data source location	Department of Biology, Faculty of Science, Universiti Putra Malaysia. (3.0017913364380053, 101.70478423864024)
Data accessibility	Raw reads were deposited to NCBI Sequence Read Archive under the Bioproject PRJNA664826. (https://www.ncbi.nlm.nih.gov/bioproject/PRJNA664826/) (Stage III – Accessions: SRX9171164, SRX9171165, SRX9171166; stage IV – Accessions: SRX9171161, SRX9171162, SRX9171163)

## Value of the Data

• The transcriptome dataset is useful to offer insight into the transcriptome regulation of key genes of interest that are responsible for the desired traits such as the genes involved in the biosynthesis of anthocyanins and flavonoids in roselle.
• The transcriptome dataset could benefit studies that focuses on pigment development, antioxidant activities and pharmacological research directed to roselle calyces. It could also be used as a reference data for researchers working on roselle.
• The transcriptome dataset allows for further downstream applications associated with newly constructed genetic resources such as genic SSR marker development and discovery of transcription factors.

## 1 Data Description

**Fig. 1:**
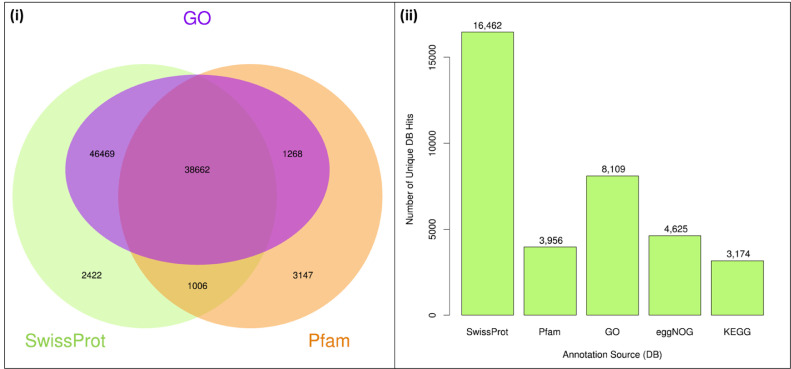
Transcript annotation results: (i) Number of transcripts annotated with SwissProt/Pfam/GO associations, and (ii) Number of unique hits database hits of annotated transcripts.

**Fig. 2:**
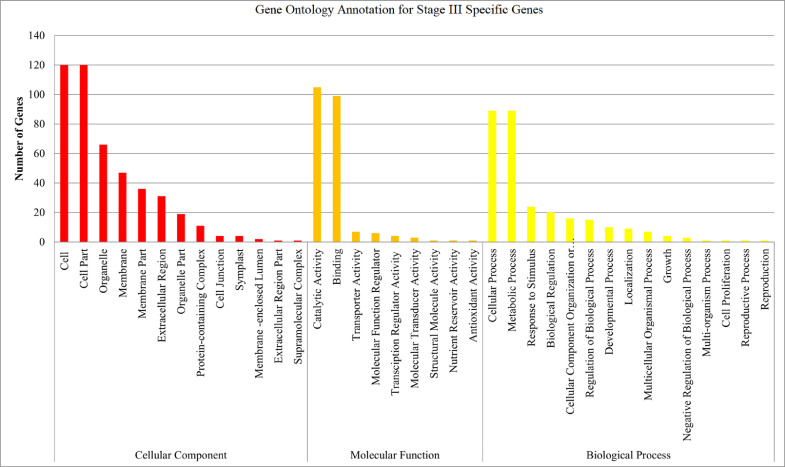
Gene ontology classification derived from stage III roselle calyx transcriptome.

**Fig. 3:**
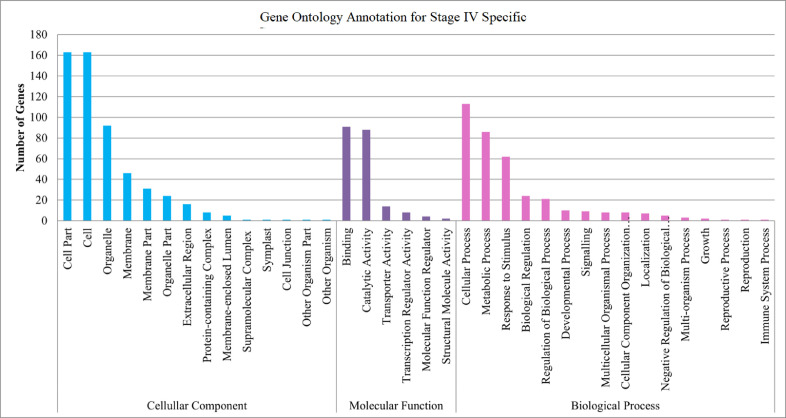
Gene ontology classification derived from stage IV roselle calyx transcriptome.

**Fig. 4:**
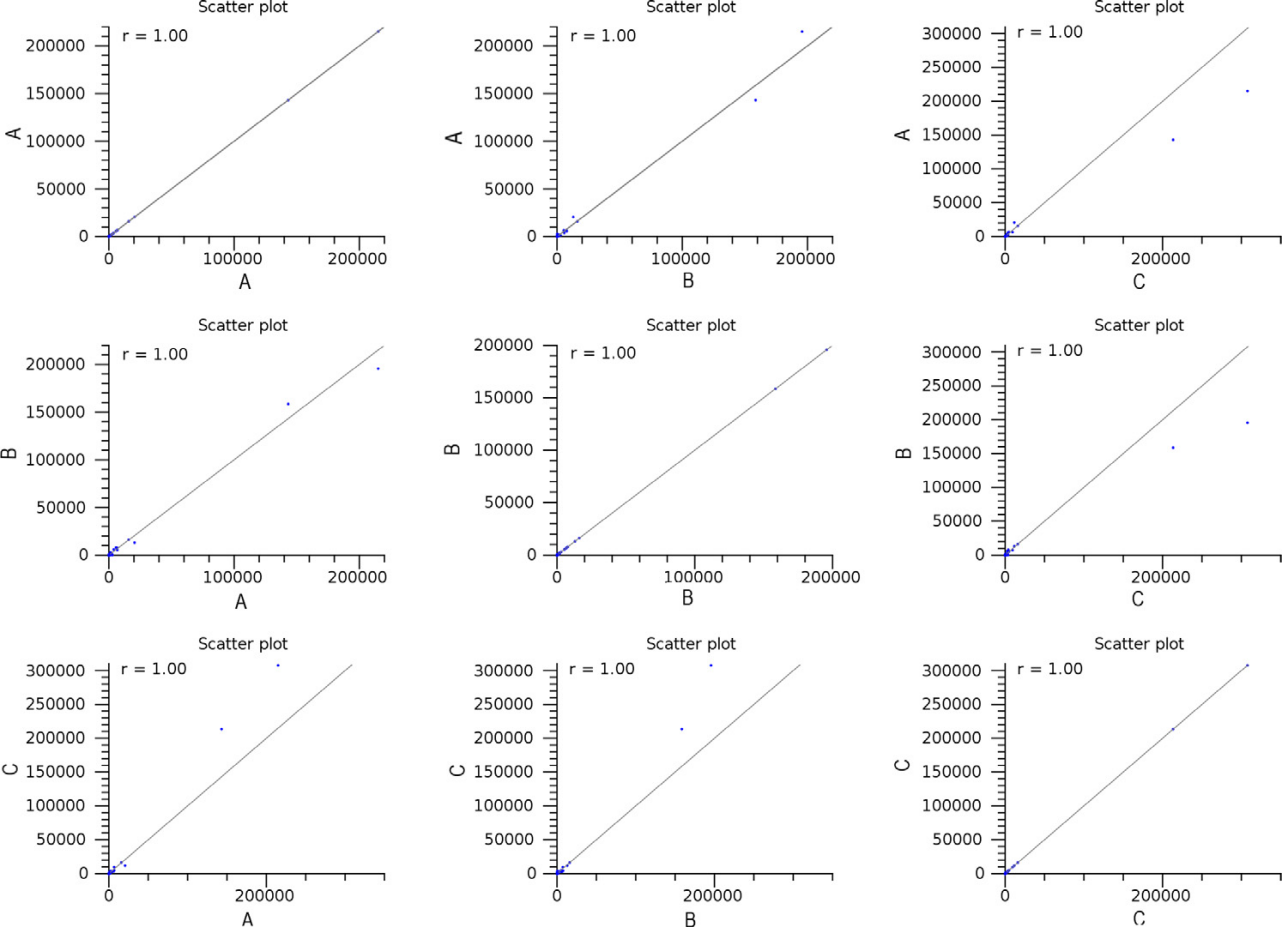
Scatter plot matrices comparing normalised expression values from biological replicates of stage III calyces.

**Fig. 5:**
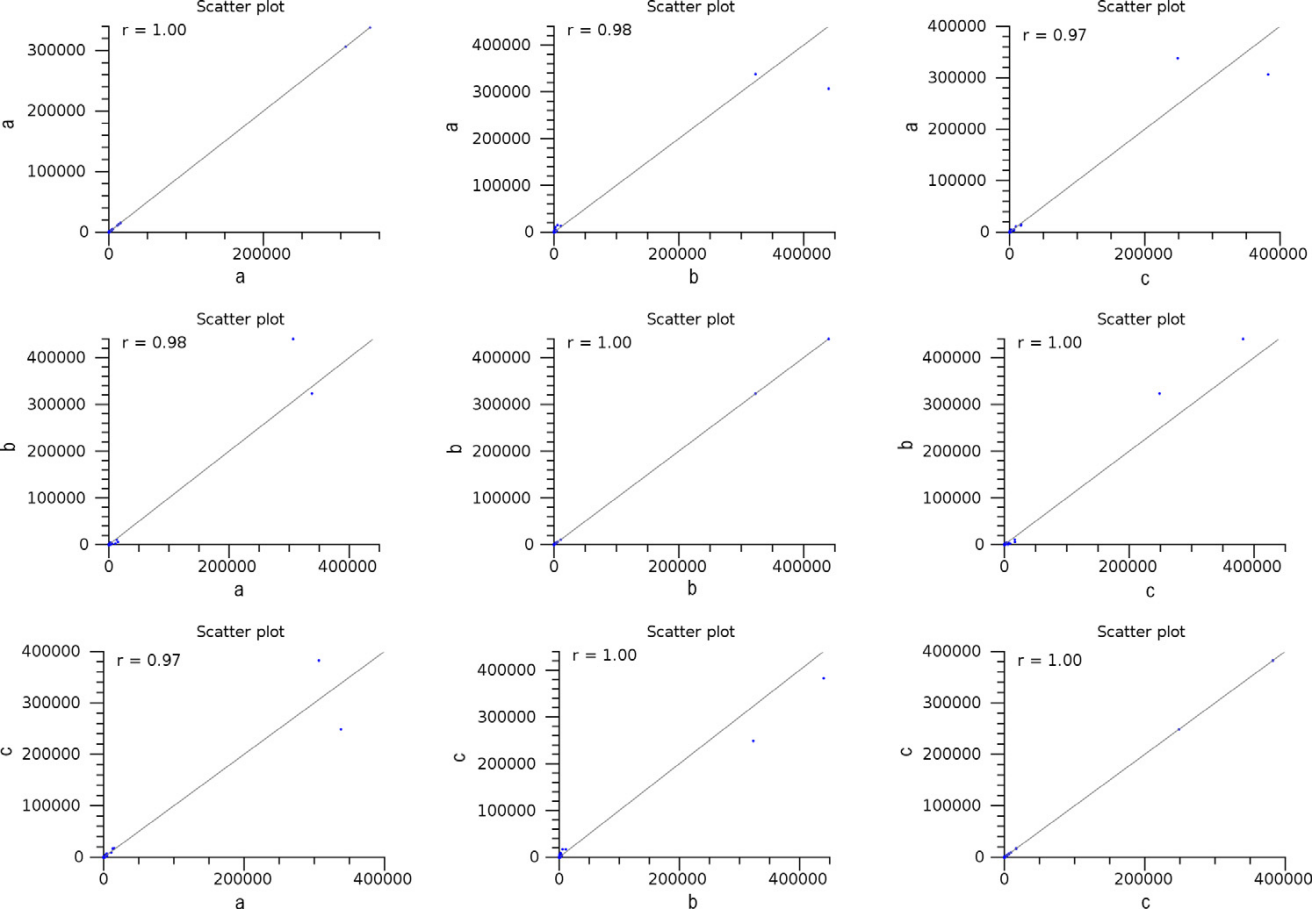
Scatter plot matrices comparing normalised expression values from biological replicates of stage IV calyces.

**Fig. 6:**
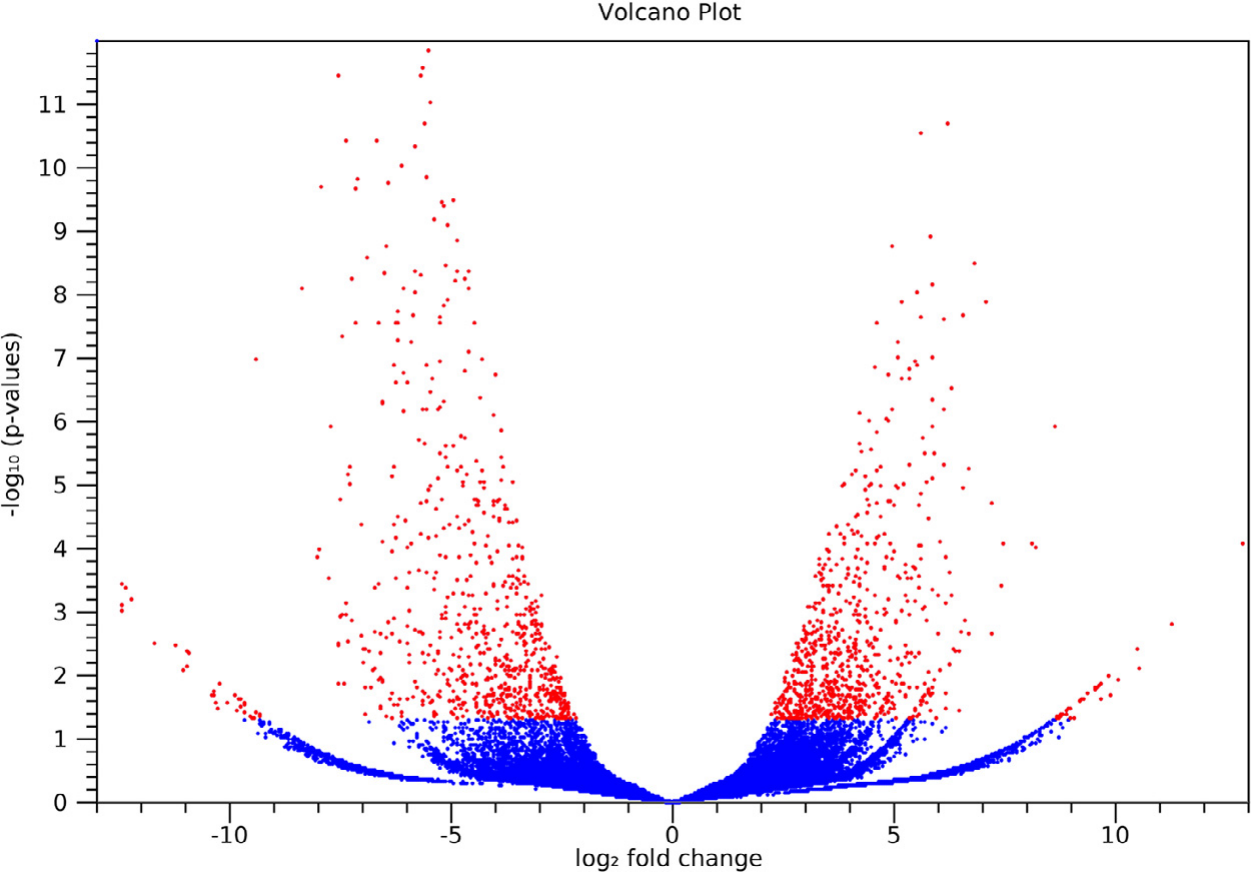
Volcano plot comparing transcripts expression in stage III and stage IV calyx tissues. The red dots represent significantly differentially expressed transcripts; whereas the blue dots represent the non-significantly differentially expressed transcripts.

**Fig. 7:**
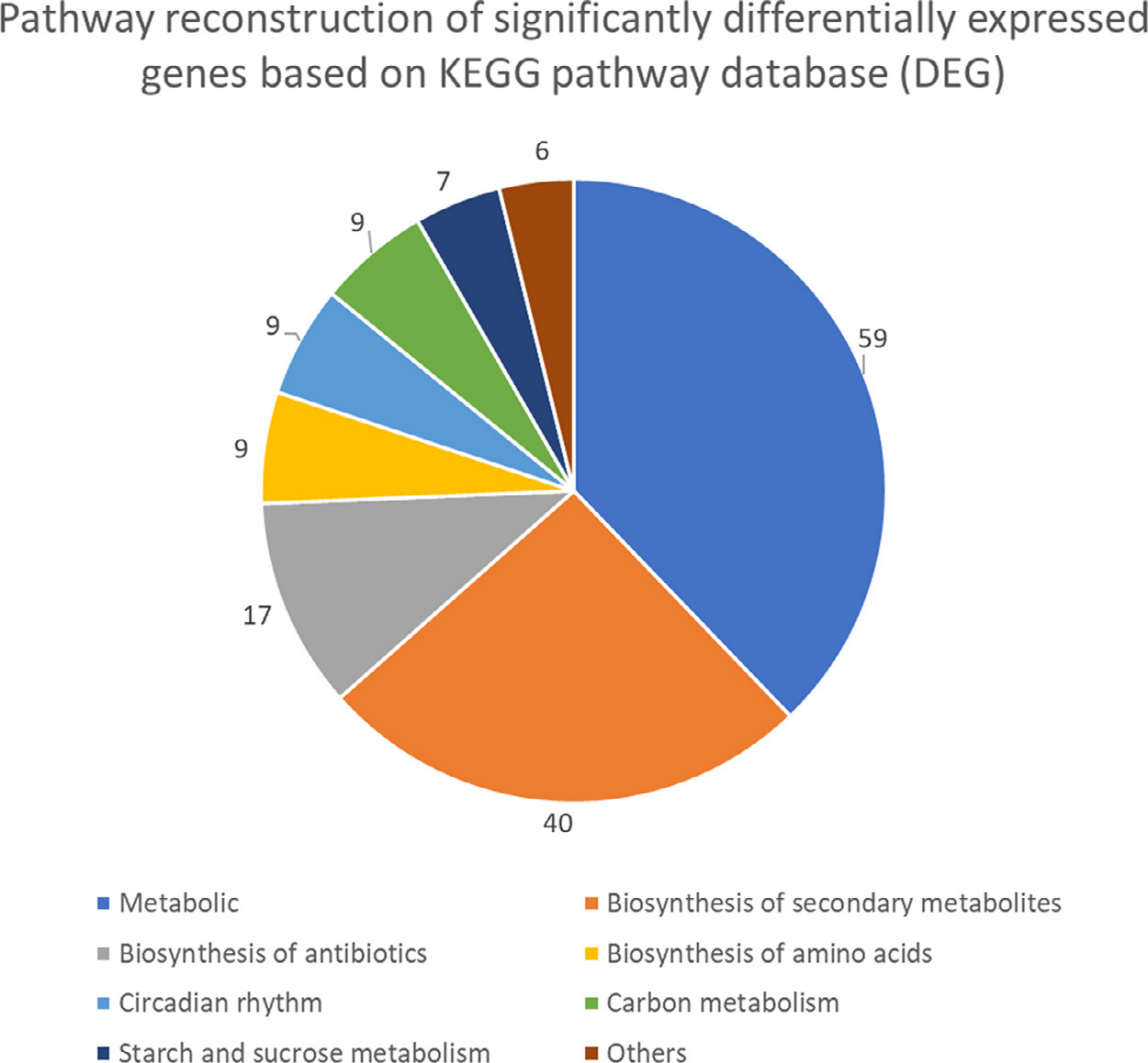
The number of significantly differentially expressed genes mapped to KEGG pathways.

The maturation stage of the calyx is commonly used as a measure to reflect its quality, where it is preferably harvested during stage III over stage IV. To date, no scientific study has been conducted to reveal the transcriptome changes in the calyces of the two stages. Six sequencing libraries prepared from stage III calyx (samples A, B, C) and stage IV calyx (samples a, b, c) were sequenced using Illumina NextSeq 500. The datasets contained raw data sequence converted to FastQ format. The raw reads were deposited into NCBI Short Read Archive with te Bioproject number PRJNA664826 (accession numbers SRX9171161, SRX9171162, SRX9171163, SRX9171164, SRX9171165 and SRX9171166). The number of total reads and good quality reads generated in this study is presented in Table 1. Transcript annotation based on SwissProt/Pfam/GO associations and the number of unique database hits are displayed in Fig. 1. Gene ontology classifications derived from stage III and stage IV roselle calyx transcriptomes are presented in Fig. 2 and Fig. 3, respectively. Fig. 4 and Fig. 5 show the scatter plot matrices comparing normalised expression values from biological replicates of stage III and stage IV calyces. Volcano plot comparing transcripts expression in stage III and stage IV calyx tissues is visualised in Fig. 6, and the total number of significantly differentially expressed transcripts between stage III and stage IV calyx tissues is summarised in Table 2. Pathway reconstruction of significantly differentially expressed genes (DEG) based on KEGG database is displayed in Fig. 7. The anthocyanin-related genes and the number of transcripts associated with them are listed in Table 3*.* The top five most significantly regulated DEGs in stage III and stage IV are shown is Table 4.

**Table 1:** Number of total raw reads, good-quality reads before and after quality trimming and filtering, and the number of low-quality reads discarded.

Sample	Raw Reads (Pairs)	Good Quality Reads (Pairs)	Low Quality Reads Discarded (Pairs)
A	63,092,989	38,507,642	24,585,347
B	65,301,544	40,661,821	24,639,723
C	65,625,766	41,267,234	24,358,532
a	61,853,842	40,981,601	20,872,241
b	59,099,055	35,605,342	23,493,713
c	65,967,482	40,298,058	25,669,424
Total	380,940,678	237,321,698	143,618,980

**Table 2:** Significantly differentially expressed transcripts between stage III and stage IV calyx tissues.

Comparison (Stage III vs Stage IV)	Number of DEG
Downregulated in Stage IV; Upregulated in Stage III	810
Downregulated in Stage III; Upregulated in Stage IV	782
Total	1,592

**Table 3:** The identified anthocyanin-related genes, their corresponding enzymes, and the number of associated transcripts in *Hibiscus sabdariffa.*

No	Gene	Enzyme	No of Transcripts
1	*TCMO*	Trans-cinnamate 4-monooxygenase [EC1.14.14.91]	7
2	*CHS*	Chalcone synthase [EC: 2.3.1.74]	15
3	*CFI*	Chalcone-flavanone isomerase [EC: 5.5.1.6]	5
4	*F3H*	Flavanone 3-hydroxylase [EC:1.14.11.9]	4
5	*FLS*	Flavonol synthase [EC: 1.14.20.6]	4
6	*F3’H*	Flavonoid 3’-monooxygenase [EC: 1.14.14.82]	7
7	*F3’5’H*	Flavonoid 3’,5’-hydroxylase [EC: 1.14.14.81]	11
8	*DFR*	Dihydroflavonol 4-reductase [EC:1.1.1.219]	10
9	*LDOX/* *ANS*	Leucoanthocyanidin dioxygenase/ Anthocyanidin synthase [EC: 1.14.20.4]	6
10	*LAR*	Leucoanthocyanidin reductase [1.17.1.3]	5
11	*UFGT/ UFOG*	Flavonoid 3-*O*-glucosyltransferase/ UDP-glucose flavonoid 3-O-glucosyltransferase [EC:2.4.2.51]	10
12	*BZ1*	Anthocyanidin 3-O-glucosyltransferase [2.4.1.115]	22
13	*3MAT*	Malonyl-coenzyme A: anthocyanin 3-O-glucoside-6′'-O-malonyltransferase/ anthocyanin 6′'-O-malonyltransferase [EC:2.3.1.171]	8
14	*5MAT*	Malonyl-CoA:anthocyanidin 5-O-glucoside-6′'-O-malonyltransferase [EC:2.3.1.172]	3
15	*5AT*	Anthocyanin 5-aromatic acyltransferase/anthocyanin 5-(6′''-hydroxycinnamoyltransferase) [EC:2.3.1.153]	8
16	*3AT*	Coumaroyl-CoA:anthocyanidin 3-*O*-glucoside-6′'-*O*-coumaroyltransferase/ Anthocyanidin 3-O-glucoside 6"-O-acyltransferase [EC:2.3.1.215]	14
17	*3GT*	Anthocyanin 3’-O-beta-glucosyltransferase/ Delphinidin 3,’5’-di-O-glucoside 3’-O-glucosyltransferase [EC:2.4.1.238]	2
18	*DUSKY/ 3GGT*	Anthocyanidin 3-*O*-glucoside 2′'-*O*-glucosyltransferase [EC:2.4.1.297]	4
19	*UGT75C1*	Anthocyanidin 3-O-glucoside 5-O-glucosyltransferase 2 [EC:2.4.1.298]	1
20	*AA5GT*	Cyanidin 3-O-glucoside 5-O-glucosyltransferase [EC:2.4.1.299]	1
21	*AA7GT*	Cyanidin 3-O-glucoside 7-O-glucosyltransferase (acyl-glucose) [EC:2.4.1.300]	1

Total:			148

**Table 4:** The top five most significantly regulated DEGs in stages III and IV.

Regulation	DEG
Upregulated in Stage III	Protochlorophyllide reductase A (*PORA*) Two component response regulator-like (*APRR5*) Peroxidase 52 (*PER52*) Thiamine thiazole synthase 2 (*THI1*-2) Chaperonin 60 subunit beta 4 (*CPN60B4*)

Downregulated in Stage IV	15.7 kDa heat shock protein (*HSP15.7*) Protein phosphatase 2C 75 (*AHGI*) Heat shock 70 kDa protein 14 (*HSP70.14*) Calmodulin-like protein 8 (*CML8*) Serine/arginine-rich-splicing factor SR34 (*SR34*)

The two secondary metabolites biosynthesis pathways that attained a relatively higher number of DEG mappings compared to other pathways within the same category were the phenylpropanoid biosynthesis pathway and the isoquinoline and alkaloid biosynthesis pathway.

## 2 Experimental Design, Materials and Methods

### 2.1 Plant material

*Hibiscus sabdariffa* variety UMKL1 (voucher number: CY001) was used in this study. The roselle planting materials were obtained from the Department of Agriculture Terengganu, Malaysia. It is a red variety registered as ‘HS2’ by the Malaya University for national crop list under the Department of Agriculture Malaysia.

Calyx tissues were harvested at stages III and IV determined based on the number of days post-anthesis (DPA), in reference to the calyx tissue maturation index guidelines provided by the Federal Agricultural Marketing Authority, Malaysia (Table 5) [1]. Three biological replicates of the calyx tissues for each maturation stage were used in this study. Biological replicates of stage III calyces (designated as samples A, B and C) were collected on 32^nd^ DPA; whereas biological replicates of stage IV calyces (designated as samples a, b and c) were collected on 59^th^ DPA. Collected samples were immediately flash-frozen in liquid nitrogen and stored in a -80˚C until RNA extraction as a preventive measure against rapid RNA degradation. A separate set of calyx samples were harvested during sampling, observed, and dissected, and the images were captured for the evaluation of phenotypic differences.

**Table 5:** Maturation index of roselle calyx based on the number of days post-anthesis.

Maturation index	Day Post-Anthesis
Stage III	26–32
Stage IV	≥33

### 2.2 RNA extraction, rRNA-depletion, cDNA sequencing library construction and RNA sequencing

Total RNA was extracted from calyx tissue samples using RNeasy Plant Mini Kit (Qiagen, Hilden, Germany) based on the manufacturer's protocol version 2012. RNA extractions were conducted in triplicate for calyx samples from each maturation stage. RNA quality and quantity were determined using Qubit Fluorometer 2.0 (Life Technologies Corporation, Carlsbad, USA) and NanoDrop spectrophotometer (Thermo Fisher Scientific, Wilmington, USA) for concentration assessment and Agilent 2100 Bioanalyzer (Agilent Technologies, Germany) via a Pico Chip and 1% agarose gel electrophoresis for preliminary assessment of band size and quality. RNA samples having a RIN value above seven and a concentration of more than 1 µM were selected rRNA-depletion.

RNA samples were subjected to rRNA-depletion using Terminator™ 5’-Phosphate-Dependant Exonuclease (Epicentre, Madison, USA) for the purpose of removing the abundance of ribosomal RNAs (rRNA) from the sample to ensure most of the sequencing output generated accounts for reads derived from messenger RNAs (mRNA) which is important for gene expression analysis. The resulted rRNA depleted RNA samples were assessed using Qubit Fluorometer 2.0 (Life Technologies Corporation, Carlsbad, USA) and Agilent 2100 Bioanalyzer (Agilent Technologies, Germany). rRNA-depleted samples having a concentration of more than 0.5 ng/µl and a rRNA contamination below 10% were selected to construct the cDNA sequencing libraries.

Constructions of the cDNA sequencing libraries were carried out using ScriptSeq™ v2 RNA-Seq Library Preparation Kit (Epicentre, Madison, USA) based on the manufacturer's handbook to reverse transcribe the less stable RNA into a more stable molecule, complementary DNA (cDNA) that is used as the template for sequencing. The ScriptSeq cDNA libraries were quantified using Qubit Fluorometer 2.0 (Life Technologies Corporation, Carlsbad, USA) and the size distribution was assessed using the Agilent 2100 Bioanalyzer (Agilent Technologies, Germany) via a High Sensitivity DNA Chip. cDNA sequencing libraries with more than 60% of the fragment sizes falling within the 200-100 bp range were selected and quantitated using the CFX96 Touch™ Real-Time PCR Detection System (Bio-Rad Laboratories Inc, USA). Libraries were sequenced using Illumina NextSeq 500 Sequencing platform (Illumina, USA), resulted in the generation of 76 bp paired-end sequences.

### 2.3 Data analysis

#### 2.3.1 *De novo* assembly and sequence annotation

Raw sequencing reads were cleaned by filtering the PhiX (Illumina co-sequencing positive control) sequences using Bowtie2 version 2.2.3 software [2,3]. The filtered reads were then subjected to Illumina sequencing adapter removal, base quality trimming (Q≥30) and removal of short reads (≤35 base pairs) with its pair via BBDUK software [4] to generate good quality sequencing reads. *De novo* reconstruction of transcriptomes using the good quality reads was performed using Trinity version 2.2.0 software [5,6] for the assembly of contiguous sequences representing full or partial fragments of the transcriptome. Protein coding region of the assembled transcripts were then predicted using Transdecoder version 2.0.1 software, and subsequently functionally annotated using Trinotate version 3.0.1 software. Sequence similarity were searched against Swiss-Prot database [7] with BLAST version 2.2.31+ [8] for protein sequences and Pfam-A.hmm database [9] via HMMER version 3.1b2 [10] software for protein domain recognition. The sequence similarity and protein domain identification results were used to link the transcripts to eggNOG [11] for identification search of biological processes, and gene ontology [12] for the exploration and retrieval of information concerning three important components: biological process, molecular function, and cellular component. The generated functionally annotated reference transcriptome was then integrated into SQLite database. Raw reads were deposited to NCBI Sequence Read Archive under the Accession PRJNA664826.

#### 2.3.2 Transcript expression analysis

Transcript expression analysis was carried out using the CLC Genomic Workbench (Version 10.1.0). Raw reads were subjected to quality control (PhiX and adapter sequences filtering, base quality trimming Q≥20, read length ≥30bp) and the good quality reads of each sample were mapped to the *de novo* assembled transcripts. Transcript abundance for every transcript was quantified in transcripts per million (TPM). Expression profiles of biological replicates were first examined to ensure the well correlation of replicates and to probe relationship among samples. Scatter plots and Pearson correlation coefficient graphs were constructed between biological replicates within samples of each stage to measure their interrelationship. Principal component analysis (PCA) was performed using RStudio IDE (version 4.1.2.) software [13] to emphasize variations and bring out strong patterns in the dataset. Pair-wise differential expression analysis was performed on the six samples. Transcripts having a fold change value (FC) of ≥2 differences and an adjusted *p*-value (FDR) of ≤0.05 were defined as significantly differentially expressed genes (DEGs) and further utilised to form a volcano plot to visualize and highlight the degree of which genes are significantly differentially expressed. A Venn diagram was constructed via Interactivenn software [14] to illustrate the distribution of DEGs between stages. An overall, comprehensive annotation bar chart was further established for each group of staged specific transcripts via WEGO 2.0 software [15,16].

#### 2.3.3 Kyoto encyclopedia of genes and genomes (KEGG) pathway analysis

**Fig. 8:**
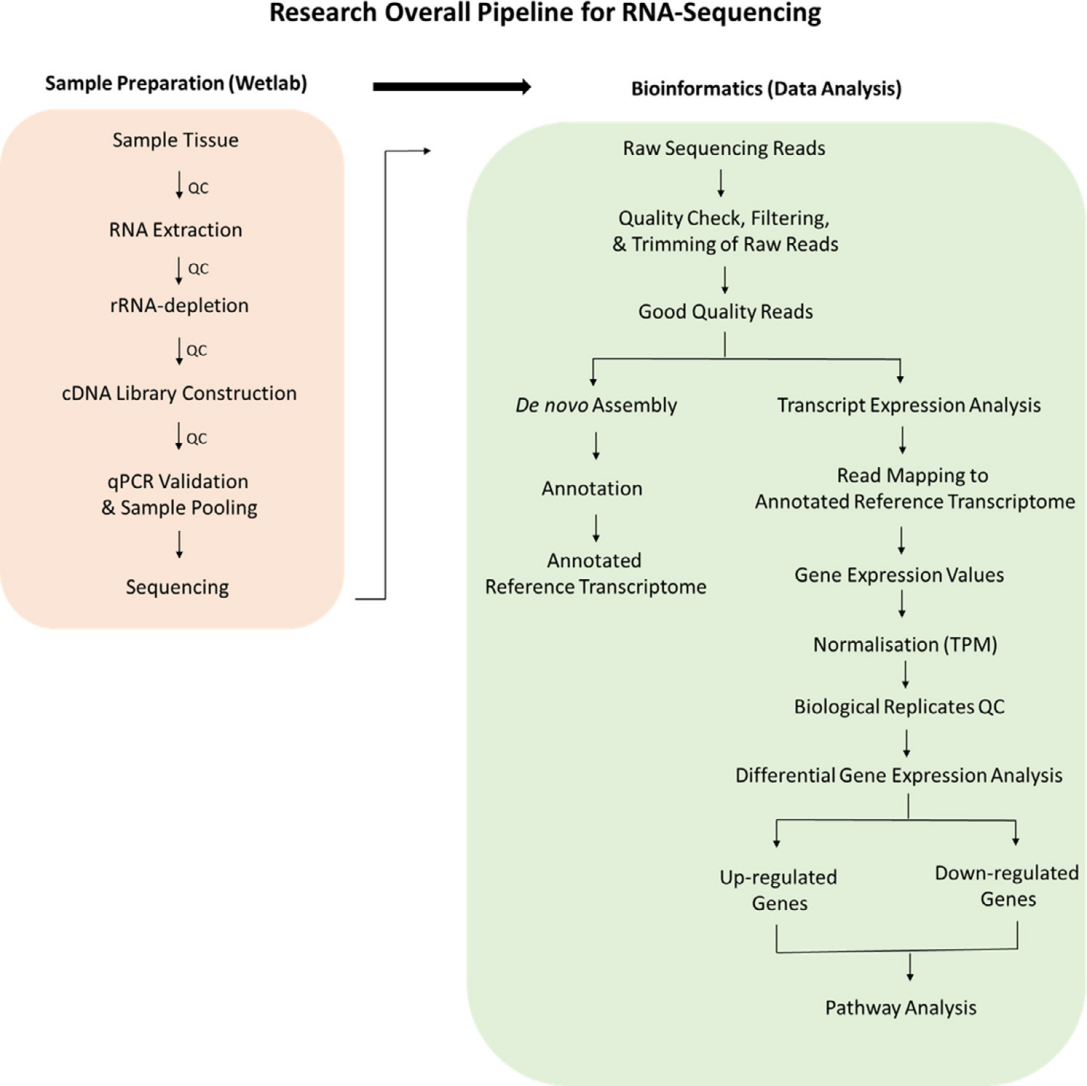
Research overall pipeline for RNA-Sequencing.

The KEGG Mapper – Search and Color Pathway software was employed for pathway analysis. Significantly differentially expressed transcripts identified from the differential expression analysis were used to obtain a list of Trinotate-annotated K numbers, which were then used for pathway reconstruction using the Kyoto Encyclopedia of Genes and Genomes web server against the KEGG pathway database. The overall flow chart of the RNA-sequencing pipeline used in this study is summarised in Fig 8.

## Ethics Statements

This study does not involve human participants and samples derived from human. It also does not involve animals including live vertebrates and higher invertebrates.

## CRediT Author Statement

**Nur Atheeqah Hamzah:** Investigation, Methodology, Formal Analysis, Validation, Writing – original draft, Visualization; **Christina Seok Yien Yong:** Conceptualization, Resources, Writing – review & editing, Visualization, Supervision, Project Administration, Funding acquisition; **Meenakshii Nallappan:** Supervision, Writing – review & editing.

## Declaration of Competing Interest

The authors declare that they have no known competing financial interests or personal relationships that could have appeared to influence the work reported in this paper.

## Data Availability

Transcriptome profiling of Hibiscus sabdariffa L during calyx maturation (Original data) (NCBI SRA database).
